# Accuracy of radiographic measurement techniques for the Taylor spatial frame mounting parameters

**DOI:** 10.1186/s12891-021-04084-0

**Published:** 2021-03-18

**Authors:** Jan Gessmann, Sven Frieler, Matthias Königshausen, Thomas A. Schildhauer, Yannik Hanusrichter, Dominik Seybold, Hinnerk Baecker

**Affiliations:** Department of Trauma Surgery, BG University Hospital Bergmannsheil, Ruhr University Bochum, Bürkle-de-la-Camp-Platz 1, 44789 Bochum, Germany

**Keywords:** Taylor spatial frame, Hexapod, Mounting parameter, Deformity correction, Planning

## Abstract

**Aim:**

The correction accuracy of the Taylor Spatial Frame (TSF) fixator depends considerably on the precise determination of the mounting parameters (MP). Incorrect parameters result in secondary deformities that require subsequent corrections. Different techniques have been described to improve the precision of MP measurement, although exact calculation is reportedly impossible radiologically. The aim of this study was to investigate the accuracy of intraoperative and postoperative radiographic measurement methods compared to direct MP measurement from TSF bone mounting.

**Methods:**

A tibial Sawbone® model was established with different origins and reference ring positions. First, reference MPs for each origin were measured directly on the frame and bone using a calibrated, digital vernier calliper. In total 150 MPs measured with three different radiographic measurement techniques were compared to the reference MPs: digital radiographic measurements were performed using soft-copy PACS images without (method A) and with (method B) calibration and calibrated image intensifier images (method C).

**Results:**

MPs measured from a non-calibrated X-ray image (method A) showed the highest variance compared to the reference MPs. A greater distance between the origin and the reference ring corresponded to less accurate MP measurements with method A. However, the MPs measured from calibrated X-ray images (method B) and calibrated image intensifier images (method C) were intercomparable (*p* = 0.226) and showed only minor differences compared to the reference values but significant differences to method A (*p* < 0,001).

**Conclusion:**

The results demonstrate that MPs can be accurately measured with radiographic techniques when using calibration markers and a software calibration tool, thus minimizing the source of error and improving the quality of correction.

## Introduction

**Fig. 1:**
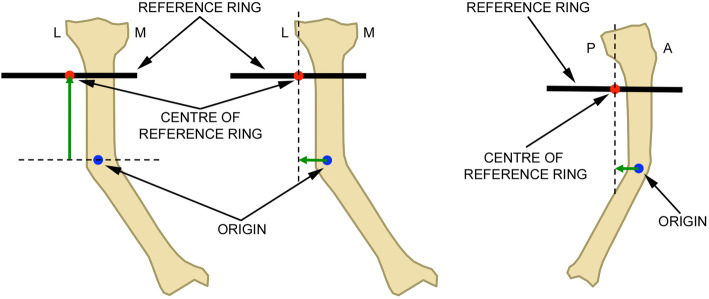
Axial, mediolateral and anteroposterior mounting parameter measurement: the green arrows mark the distances between the origin (blue dot) and the centre of the reference ring (red dot). Each distance defines the respective MP for the axial, mediolateral and anteroposterior frame offset in relation to the origin. L: lateral; M: medial; P: posterior; A: anterior

The Taylor Spatial Frame (TSF) is an external hexapod fixator that allows simultaneous correction of deformities in all six axes. This is achieved by the movement of six telescopic struts connected to two rings that rotate and translate the bone segments around a virtual hinge. The location of the virtual hinge, known as the origin, can be freely defined by the surgeon and is usually positioned at the apex of the bony deformity [1]. The complex movements of the 6 telescopic struts are calculated by the web-based TSF software application that generates a schedule for the daily strut adjustments. The accuracy of the calculation and thereby the correction result depends on accurate deformity parameters, frame parameters and mounting parameters (MPs). The latter define the location of the virtual hinge relative to the TSF reference ring (Fig. 1) [1]. Although inadequate frame and deformity parameters also result in residual deformity, the MPs have been identified as the main source of error [1–5]. Various intra- and postoperative measurement techniques and technical tricks have been proposed to facilitate accurate determination of the MPs and prevent residual corrections [1, 3–7]. However, many patients still require adjustments after correction due to residual deformities, and up to five schedules (average 2.5), with an X-ray follow-up required for each rescheduling, have been reported in the literature, which is consistent with our own experience [7–12]. Although the TSF allows further residual corrections without reassembling the frame or redefining inaccurate initial MPs, which is one of its major benefits [1], rescheduling lengthens the duration of treatment and the time spent in frame and may therefore also reduce patient compliance; the number of repeat X-ray follow-ups is minimized if MPs are accurately determined [3]. Originally, MPs were obtained from postoperative anteroposterior (ap) and lateral radiographs with a TSF grid on hard-copy images, but they are now usually measured on digital images using software measurement tools or measured intraoperatively directly from the patient frame [1, 5]. Measuring directly on the frame and bone fragments provides the most accurate parameters, but patient factors such as extensive soft tissue may impede direct measurement in clinical practice. However, exact MP calculation is reportedly impossible with radiographic techniques [3]. To our knowledge, no study has compared radiographic measurement techniques to the gold standard of direct measurement on the frame and bone. Therefore, we conducted this study to analyse the accuracy of postoperative and intraoperative radiographic measurement methods against direct measurement from the TSF mounted on a Sawbone® model. We sought to determine whether a surgeon can rely on radiographic measurement techniques as much as on direct measurement and –if any differences exist- which technique is the most precise.

## Methods

To compare the different radiographic methods with direct measurement for defining MPs, a tibial Sawbone® model was established. Two 180-mm TSF rings were applied under fluoroscopic control in strict orthogonal alignment and without rotation to the mechanical axis of the bone in both coronal and sagittal planes with 6-mm half pins, thus allowing both rings to be used as possible reference rings. The position in zero degree rotation was important because a radiological determination of the rotation on two-dimensional X-ray images is not possible and can only be measured clinically or with a CT scan [3]. The different origin positions were defined by sticking small steel balls (1 mm in diameter) to the Sawbone® model as radiopaque markers. Altogether, six different TSF bone models were assembled with 50 different origin positions. Each origin position was defined by three MPs.

The MPs for axial, anteroposterior (ap) and mediolateral (ml) distances between each origin and reference ring for each mounted TSF were then determined with the use of a calibrated, digital vernier calliper (MMO, Germany, model 3500, measurement accuracy 0.02 mm). The obtained 150 MPs were defined as reference MPs to which the different radiographic measurement techniques were compared. Then, ap and lateral ring markers were attached to the rings using threaded rods or bolts on each side. One ap ring marker was attached to the centre hole of the master tab and another was attached to the spare hole on the opposite side of the ring. For the lateral ring markers, 30-mm bolts were placed through the centre holes of the medial and lateral aspects of the ring, 180° across from each other.

Three different digital measurement techniques were used for radiographic determination of the MPs, including measurements performed from soft-copy PACS images without (method A) and with (method B) calibration and from calibrated image intensifier images (method C).

**Fig. 2:**
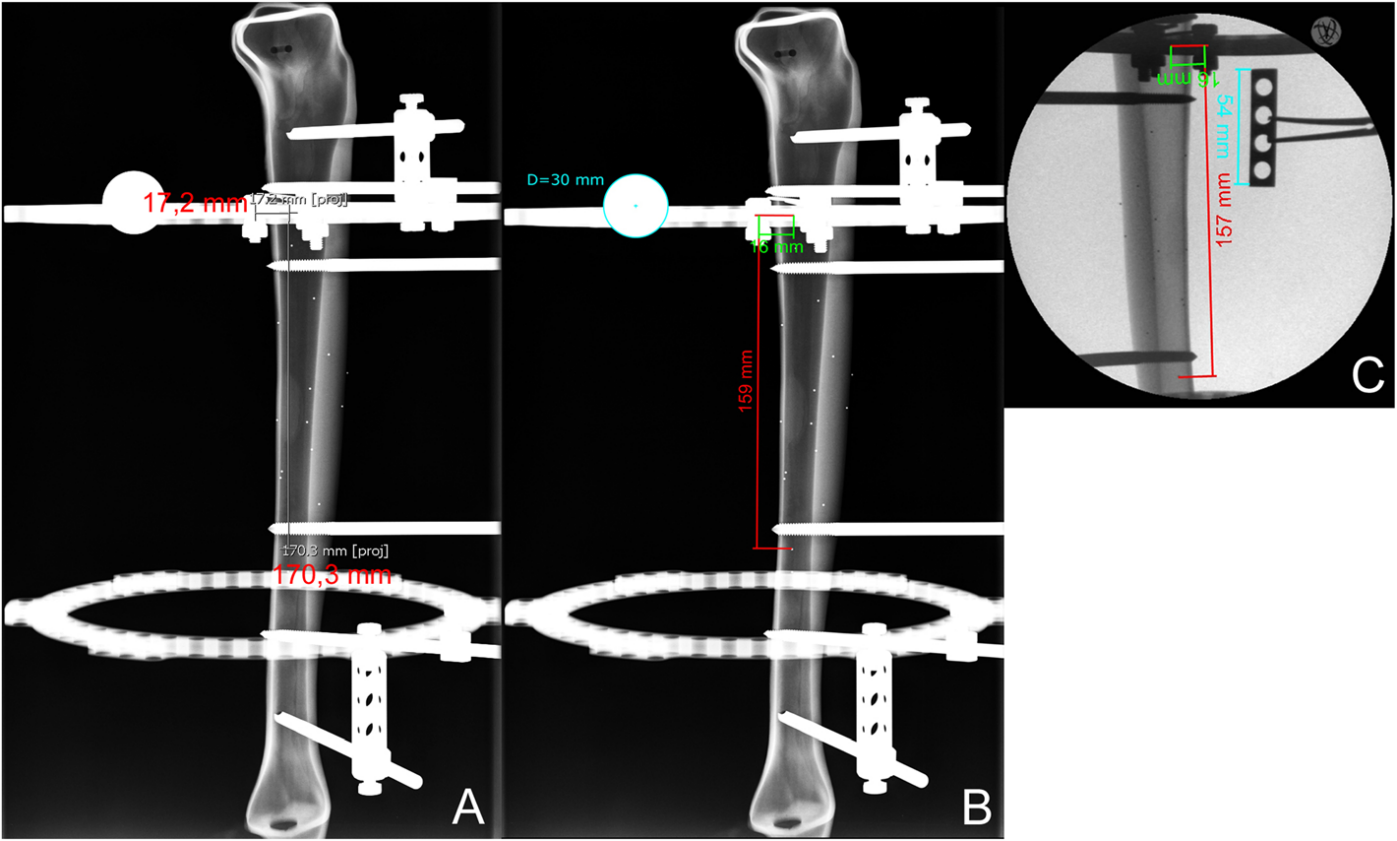
Lateral view for axial and anteroposterior MP measurements showing the superimposed bolts on an X-ray image without (**a**, method A) and with (**b**, method B) calibration as well as on a fluoroscopic intensifier image (**c**, method C); the axial MP on X-ray image **a** (170 mm) shows high deviation compared to those on calibrated X-ray image **b** (159 mm) and fluoroscopic image **c** (157 mm), whereas the anteroposterior MP shows only a minor deviation of 1 mm (**a**: 17 mm, **b** and **c**: 16 mm)

The focus-film distance was 115 cm in all cases of method A and B. The focus-object distances were not documented. For digital calibration of the X-ray picture, a 30-mm calibration ball was placed at the level of the bone and close to the reference ring. Radiographs were then taken in an orthogonal plane relative to the reference ring with the X-ray beam centred on the ap (master tab) or lateral marker. Adequate radiographs for MP determination required the ring to be rendered as a flat line as well as overlapping of the two corresponding ap or lateral markers (Fig. 2).

The “intraoperative” technique using a fluoroscopic image intensifier was performed following a technique similar to that described by Gantsoudes et al. [1] and Park and Bradish [4]. Anteroposterior and lateral orthogonal views were obtained by rotating the Sawbone® under fluoroscopy until the reference ring was rendered as a flat line and the ap or lateral markers were exactly superimposed. As a calibration marker, a four-hole rancho cube was held with a clamp at the bone level and close to the reference ring for subsequent calibration of the digital image, ensuring that the rancho cube was displayed perpendicular with all holes appearing as circles under fluoroscopy (Fig. 2).

MPs were then digitally measured using either the measuring tool of the PACS software on a non-calibrated digital X-ray-image (method A) or using the mediCAD® (HECTEC, Germany) software application with calibrated images using either the 30-mm calibration ball (method B) or the four-hole-rancho cube on the intensifier images (method C), respectively. All 150 reference MPs were then digitally measured with each of the three radiographic methods resulting in a total of 450 measured values. All measurements were performed by surgeons with long-term TSF experience.

All statistical analyses were performed using commercial statistical software (Statistical Package for Social Sciences Software, IBM SPSS Statistics Version 24, Chicago, IL). Differences in proportions were compared using ANOVA analysis. To determine intercomparability a Bonferroni post-hoc analysis was conducted. Significance level was set as *p* < 0.05.

## Results

The mean results of the MPs as well as the mean differences of the measured values determined by the three different digital measurement methods compared to the those determined by the reference method are summarized in Tables 1 and 2. Axial, ap and ml MPs measured on a non-calibrated X-ray image (method A) showed the highest variance compared to the reference MPs. However, the highest differences were observed in the measured axial MPs, with a mean deviation of 11 (1–21) mm. The largest deviations of up to 21 mm compared to the reference measurements were found at the furthermost axial offset of the origin relative to the reference ring (140 mm). The ap and ml MPs measured with method A differed by an average of 1.3 mm and 2.7 mm, respectively. The most precise values were measured with method B, with differences of only 0.7 (0–2) mm for axial measurements and 0.2 (0–1) mm for ap measurements and an average deviation of 0.5 (0–1) mm compared to the reference measurements. The values measured with method C were nearly as precise as those measured with method B: the axial MPs differed by 1.8 (0–4) mm on average, the ap MPs differed by 0.4 (0–1) mm and the ml MPs differed by 1.5 (0–3) mm.

**Table 1:** Mean axial, antero-posterior (ap) and medio-lateral (ml) mounting parameters in mm (ranges in brackets) determined by a Vernier caliper (reference) and the three different digital measurement methods

	Reference	Method A	Method B	Method C
axial MP	86.6 (15–144)	97.6 (16–164)	86.5 (15–145)	86.4 (16–142)
ap MP	9.6 (0–19)	10.9 (0–21)	9.5 (0–18)	9.4 (0–18)
ml MP	23.1 (10–42)	25.8 (11–47)	23.0 (9–43)	21.6 (8–40)

**Table 2:** Mean differences in mm (ranges in brackets) of the measured values determined by the three different digital measurement methods compared to those measured using the reference method

	Method A	Method B	Method C
axial MP	11 (1–21)	0.7 (0–2)	1.8 (0–4)
ap MP	1.3 (0–3)	0.2 (0–1)	0.4 (0–1)
ml MP	2.7 (1–6)	0.5 (0–1)	1.5 (0–3)

The ANOVA analysis indicated significant differences between the individual methods (*p* < 0.001, eta-square = 0,363). The Bonferroni post-hoc analysis revealed significant differences between method A and method B (*p* < 0.001) as well as method A and method C (*p* < 0.001). The measurements from the calibrated X-ray images (method B) and the calibrated image intensifier images (method C) showed only minor and non-significant differences (*p* = 0,226).

## Discussion

Hexapod-based ring fixators such as the TSF have simplified deformity correction due to their modularity together with web-based planning software. The high theoretical accuracies of 1/1,000,000 in. and 1/10,000 degrees are extreme for clinical practice, but with approximate correction accuracies of 1 mm and 1°, the TSF is a very powerful device for deformity correction [1]. However, the TSF can only provide accurate corrections if the MPs are measured and programmed precisely [1, 5]. Even with extensive surgical experience with the frame accurate MP measurements can be difficult, and slight errors result in residual deformity in angulation and/or translation requiring subsequent correction [1, 13, 14]. Although one of the major benefits of the TSF is the ability to conduct residual corrections without reassembling the frame, the consequences are treatment delay, longer healing indices and more follow-up X-rays.

The quality of radiographic MP determination is essentially associated with the quality of the ap and lateral X-ray images, which must be perfectly orthogonal to the plane of the reference ring to display it as a flat line on the image [7]. The clinical problem with the postoperative radiographic technique is that it relies on precise positioning of the central X-ray beam on the exact orthogonal plane of the reference ring. In our “in vitro” study, obtaining precisely adjusted X-rays of Sawbone® frame-mounting was not difficult. However, in clinical practice, accurate imaging can be problematic depending on factors such as patient positioning with frame assembly and the exact adjustment technique of the radiologist [5–7]. Therefore, Kanellopoulos et al. [5] proposed a custom-made radiolucent guide frame that can be attached to the TSF to guide the surgeon and radiologist in obtaining radiographs that are perfectly perpendicular to the reference ring [5]. The authors found that the guide frame was very useful in children and adolescents, but its application may be limited in obese adults and in patients who cannot tolerate the necessary positioning [5]. Recently, Wright et al. [7] proposed the “silhouette technique”: The authors used a shadow silhouette created by the reference ring projected as a single line to indicate an orthogonal view. For MP measurements, the entire reference ring must be depicted on the X-ray image, and the orientation and field view must be indicated to the radiologist by stickers applied on the reference ring before the X-ray. In all eight patients, the authors obtained adequate radiographs on which planning of the mounting parameters was possible [7]. In contrast to MP measurements on postoperative X-rays, Gantsoudes et al. [1] and Park and Bradish [4] used intraoperative fluoroscopic intensifier images. Therefore, image quality is the responsibility of the surgeon regardless of postoperative imaging. The frame is rotated as long as the reference ring is depicted as a flat line with the ap or lateral markers exactly superimposed.

**Fig. 3:**
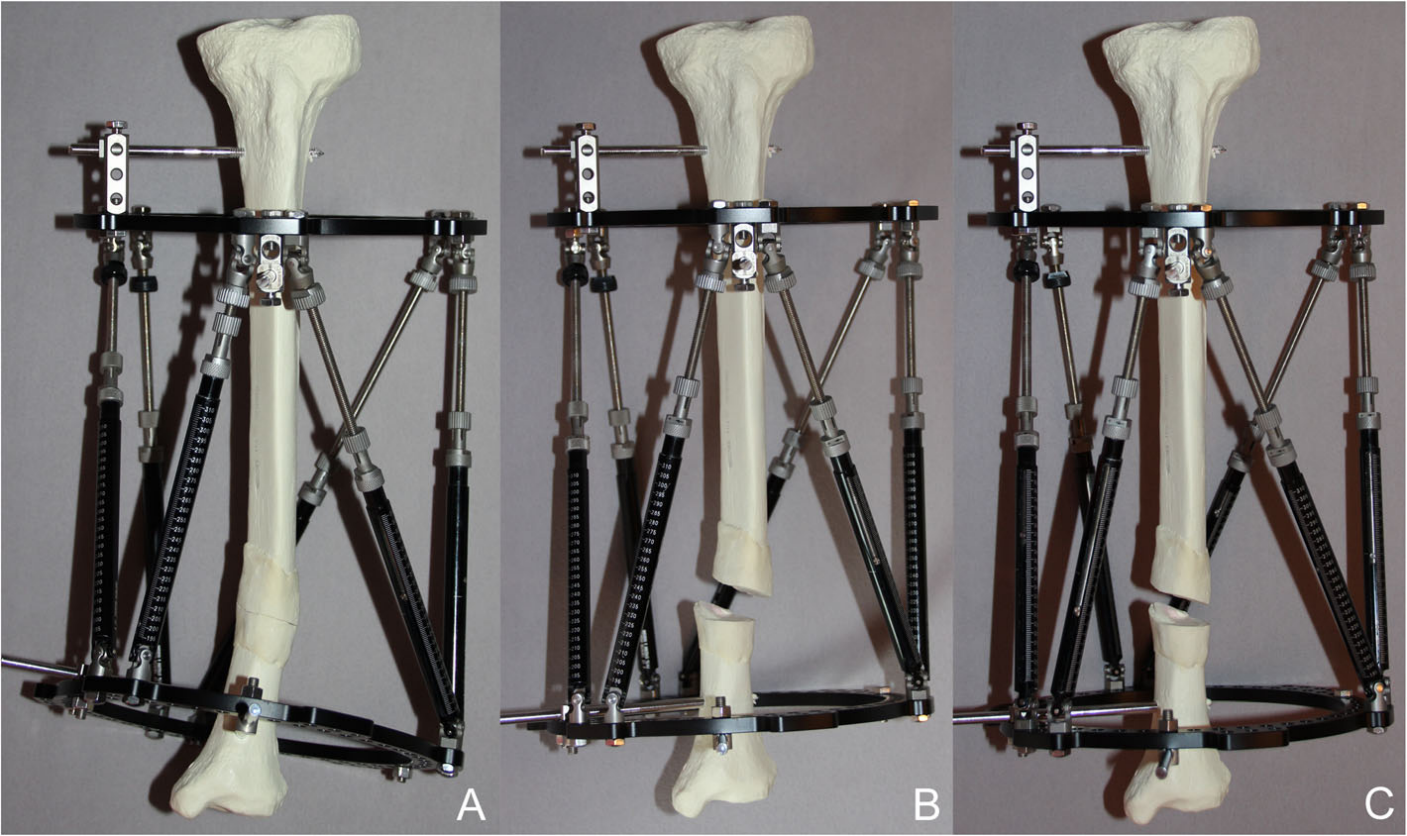
Example of a proximal reference ring attached at a substantial axial distance from a distal varus deformity (**a**); secondary medial translational deformity (**b**) after correction with MP determination from a non-calibrated image; correct axis alignment (**c**) after MP determination from a calibrated image

In addition to the need for exact orthogonal images, all radiographic and intraoperative fluoroscopy techniques must consider magnification effects. Gantsoudes et al. [1] used an image intensifier to obtain exact orthogonal images but measured MPs with a sterile ruler directly off the frame and rancho cube instead of intensifier images. The authors recognized the magnification problem due to the offset of the rancho cube from the bone, although they reported that this is negligible as long as the origin is close to the reference ring [1]. However, a greater distance between the reference ring and the origin results in less accurate MP measurements (Fig. 3). Therefore, the ring mounted closest to the origin is usually defined as the reference ring [1]. To account for the magnification effect, Park and Bradish [4] proposed the use of a calibration ball on the intensifier images. With their technique, MPs are measured on a printed image, and the surgeon adjusts the values for the magnification factor by calculation. Kucukkaya et al. [3] described a CT-based technique for precise calculation of the MPs and proposed that exact calculation is impossible with radiographic techniques. However, our results demonstrate that this only applies to non-calibrated images. With calibrated X-ray and fluoroscopic images, magnification problems can be minimized as MP measurements, even at significant axial distances, are comparable to reference measurements in our study. The calibration tool used in our study simplifies the technique by automatically calculating the magnification effect. The image can be standardized to a known dimension and the magnification is calibrated to the size of the calibration marker. The measurement scale on the X-ray images is then based on the calibration tool of the planning software [15]. On non-calibrated images, the unknown extent of magnification results in higher measured values. While the magnification effect is relatively small at short distances, it increases proportionally with longer distances. This is the reason for the large differences at the furthermost distances of the axial MPs measured on non-calibrated images in our study (method A), whereas the ap and ml MPs showed significant but smaller absolute differences. The clinical impact of the smaller MP differences may be negligible as Gantsoudes et al. [1] proposed, but the extent to which an MP miscalculation or a residual or new deformity becomes clinically evident is unknown. In a clinical trial, Sokucu et al. [16] compared intraoperative versus postoperative MP measurements in 17 cases. The authors measured the parameters under fluoroscopy during surgery as described by Gantsoudes et al. [1] and compared the values to measurements taken from digital postoperative radiographs. In their report, whether the X-ray images were calibrated is not mentioned, and the measured MPs that were used for the actual correction plan are not indicated. Although the authors reported that they found no significant differences between the intraoperative and postoperative MPs, the compared values showed substantial variance, particularly the axial MP values: 95.3 (range: 25 to 155) mm intraoperatively versus 109.5 (range: 28 to 195) mm postoperatively [16], indicating that the greatest difference of a single MP value measured with the two techniques was 40 mm (155 versus 195). This obvious inaccuracy must result in new deformities during correction and lengthening and was very likely caused by magnification effects. In a bone model Kucukkaya et al. [3] showed for example that already a 10 mm-error in the MP measurements caused a residual translation of 7 mm during the correction of a 10° rotational deformity.

The difficulty of determining the correct magnification factor of radiographs for digital planning is a major problem and still of ongoing debate in orthopaedic surgery [17]. One of the major challenges in clinical practice is the correct positioning of the calibration markers [17, 18]. A structural measurement error may occur if the position of the calibration marker deviates too much from the plane of interest [17, 19]. This is a major limitation of our study as the markers (calibration ball or rancho cube) were positioned at the bone level and not at the exact level of the different virtual hinges (the respective steel balls). The extent of a potentially structural error in calibration cannot be discussed in our study as various MPs were determined on X-ray pictures without changing the position of the calibration marker (Fig. 2). In digital planning of hip arthroplasties, for example, a mean error of 6% and range from − 5 to 15% has been described despite the use of calibration markers [20]. Other studies found absolute differences between the actual and calculated size ranged from 0.16 to 1.40 mm [17, 21–23]. The clinical difficulties of positioning radiological markers are related to patient specific factors (e.g., obesity and/or bony deformities) [17]. In patients with external TSF-frames it is found to be difficult or sometimes might even be impossible to install a calibration marker at the correct plane as it may interfere with the circular rings, soft tissues, half pins and thin wires.

Another limitation is the requirement of positioning the reference ring in zero degree rotation, only controlled by eyeballing and not verified using a CT scan in our study. Therefore, the effects on MP measurements of any possible malrotation of the reference ring cannot be discussed. However, the TSF software allows for a non-orthogonal mounting of the reference ring only in the axial plane: this is the reference ring being mounted in internal or external rotation [24]. In clinical practice a rotatory frame offset is usually determined by approximation and therefore avoided as far as possible [3, 9, 10].

In our clinical practice, we have been using the intraoperative fluoroscopic technique, referencing images with a rancho cube at the bone level (method C). With calibrated digital images, a surgeon can define the origin postoperatively at any time and double-check plans and calculations with colleagues, enabling recalculations that may be required in rare cases during treatment. One disadvantage of this technique is the increased fluoroscopic use required to obtain adequate intraoperative images perpendicular to the reference ring and to the rancho cube. On the other hand, the surgeon controls the image quality independently, without requiring radiologists and subsequent postoperative X-rays to obtain orthogonal images of the reference ring. The slight deviation of the measured MP values relative to the direct measurements may be due to incomplete orthogonal rendering of the reference ring as it is only depicted partially on the intensifier images. Another reason for the measurement deviation might be explained by the problem of image intensifier distortion that occurs with intraoperative imaging [25]. The obtained images in this study were not corrected for distortion. However, even with large axial distances between the reference ring and the origin, the maximum deviation was no more than 4 mm (or a maximum percentage difference of 3%). We have been using this technique since 2012, and the number of correction plans due to residual deformities has been reduced from up to 5 per case to a maximum of 2 per case. However, this result may be affected by many different factors, such as increased surgical experience with the TSF system over the years.

## Conclusion

In conclusion, the results of this study demonstrate for the first time that with calibrated X-ray or fluoroscopic images, TSF MPs for anteroposterior, mediolateral and axial frame offset can be measured as accurately as with direct measurement techniques. The origin can freely be controlled, defined and redefined at any time during treatment without considering greater distances from the reference ring, which may minimize the source of error and improve the quality of correction. As accurate postoperative imaging may be difficult in clinical practice we recommend the intraoperative fluoroscopic technique (method C) as the surgeon controls the quality of the image independently.

## Data Availability

The authors confirm that the data supporting the findings of this study are available within the article.
